# *In vivo* stepwise immunomodulation using chitosan nanoparticles as a platform nanotechnology for cancer immunotherapy

**DOI:** 10.1038/srep38348

**Published:** 2016-12-02

**Authors:** Hee Dong Han, Yeongseon Byeon, Jong-Hwa Jang, Hat Nim Jeon, Ga Hee Kim, Min Gi Kim, Chan-Gi Pack, Tae Heung Kang, In Duk Jung, Yong Taik Lim, Young Joo Lee, Jeong-Won Lee, Byung Cheol Shin, Hyung Jun Ahn, Anil K. Sood, Yeong-Min Park

**Affiliations:** 1 Department of Immunology, School of Medicine, Konkuk University, Chungju 380-701, South Korea; 2 Department of Dental Hygiene, Hanseo University, Seosan 31962, South Korea; 3 Department of Convergence Medicine, University of Ulsan College of Medicine & Asan Institute for Life Sciences, Asan Medical Center, Seoul 055-05, South Korea; 4 SKKU Advanced Institute of Nanotechnology (SAINT), School of Chemical Engineering, Sungkyunkwan University, Suwon 25-2, South Korea; 5 Department of Bioscience and Biotechnology, Sejong University, Kwang-Jin-Gu, Seoul 143-747, South Korea; 6 Department of Obstetrics and Gynecology, Samsung Medical Center, Sunkyunkwan University School of Medicine, Seoul 06531, South Korea; 7 Bio/Drug Discovery Division, Korea Research Institute of Chemical Technology, Daejeon 305-600, South Korea; 8 Center for Theragnosis, Biomedical Research Institute, Korea Institute of Science and Technology, Seoul 136-791, South Korea; 9 Department of Gynecologic Oncology and Reproductive Medicine, the University of Texas M.D. Anderson Cancer Center, Texas, USA; 10 Department of Cancer Biology, the University of Texas M.D. Anderson Cancer Center, Texas, USA; 11 Center for RNA Interference and Non-coding RNA, The University of Texas M.D. Anderson Cancer Center, Texas, USA

## Abstract

Dentritic cell (DC)-based cancer immunotherapy faces challenges in both efficacy and practicality. However, DC-based vaccination requires multiple injections and elaborates *ex vivo* manipulation, which substantially limits their use. Therefore, we sought to develop a chitosan nanoparticle (CH-NP)-based platform for the next generation of vaccines to bypass the *ex vivo* manipulation and induce immune responses via active delivery of polyinosinic-polycytidylic acid sodium salt (poly I:C) to target Toll-like receptor 3 (TLR3) in endosomes. We developed CH-NPs encapsulating ovalbumin (OVA) as a model antigen and poly I:C as the adjuvant in an ionic complex. These CH-NPs showed increased *in vivo* intracellular delivery to the DCs in comparison with controls after injection into tumor-bearing mice, and promoted DC maturation, leading to emergence of antigen-specific cytotoxic CD8+ T cells. Finally, the CH-NPs showed significantly greater antitumor efficacy in EG.7 and TC-1 tumor-bearing mice compared to the control (p < 0.01). Taken together, these data show that the CH-NP platform can be used as an immune response modulatory vaccine for active cancer immunotherapy without *ex vivo* manipulation, thus resulting in increased anticancer efficacy.

Nanoparticle (NP)-based active cancer immunotherapy has the potential to treat cancer without side effects^1^,^2^,^3^. A number of immunization protocols that were tested in clinical trials and are approaching clinical applications involve dendritic cell (DC)-based or adoptive T cell transfer strategies^4^,^5^. The use of DC-based therapeutic vaccines is a promising strategy against cancer and has been validated in several clinical trials^6^,^7^,^8^. Although DC-based vaccine approaches have been shown to be effective in clinical trials, they are complex and require multiple *ex vivo* manipulations beginning from isolation of DCs from the blood of patients, exposure of the DCs to antigens and other maturation stimuli, and finally reinjection of the DCs into the patients. This is a personalized but expensive therapeutic approach and time-consuming tasks^4^. Therefore, to overcome these limitations, NP-based vaccines are being studied as the next-generation platform for induction of an immune response without *ex vivo* manipulation of DCs. A suitable NP-based vaccine should provide practical advantages including simplicity, low cost of manufacturing, and potent immunogenicity.

NP-based active cancer immunotherapy has attracted interest regarding DC maturation and activation *in vivo.* These processes result in strong immunotherapeutic responses to cancer. DCs are the most effective antigen-presenting cells (APCs), which present antigens to T cells and secrete pro-inflammatory cytokines, resulting in tumor-specific activation of cytotoxic T cells through cross-presentation on major histocompatibility complex (MHC)-1 molecules^3^,^9^,^10^. Therefore, *in vivo* maturation of DCs is a key first step for effective NP-based active cancer immunotherapy. At this step, efficient delivery systems that are suitable for antigen and adjuvant delivery into DCs play a crucial role in initiating T cell mediated immunity.

Polyinosinic-polycytidylic acid sodium salt (poly I:C), is a Toll-like receptor 3 (TLR3) ligand and has shown promise as a vaccine adjuvant for a CD8+ T cell response besides, poly I:C can efficiently drive maturation of DCs, thus leading to cross-presentation of various antigens^11^. Double-stranded RNA (dsRNA) is a known TLR3 ligand and activates the TRIF dependent signaling pathway^12^. Poly I:C, a synthetic dsRNA mimic copolymer, is also a specific TLR3 ligand. Nevertheless, the therapeutic efficacy underlying the adjuvant effects of poly I:C dependent with TLR3 stimulation of a CD8+ T cell immune response have not been fully elucidated *in vivo*. Therefore, we selected poly I:C as a potent adjuvant to stimulate TLR3-mediated DC maturation. By contrast, naked poly I:C cannot penetrate the cell membrane efficiently *in vitro* and *in vivo* and is rapidly degraded by nucleases present in human plasma^13^. Therefore, an efficient system of delivery of poly I:C into DCs is urgently needed for enhancement of its immunostimulatory activity. Intracellular delivery of poly I:C using NPs through an endocytosis mechanism leads to increased entrapment of NPs in endosomes within a DC without distribution to the cytoplasm and protects poly I:C from degradation by nucleases. After intracellular uptake, poly I:C can be recognized by TLR3 in the endosome. Endosomal TLR3 is the crucial binding site for poly I:C for stimulation of an immune response. At this time, chicken egg ovalbumin (OVA) as an antigen can be released from the endosome to stimulate antigen-specific DC maturation.

Since DCs have a high capacity for antigen uptake, various nanomaterials have been developed for their specific physicochemical properties and are currently studied for their potential as drug delivery systems for immunotherapy^14^ as tools for molecular imaging^15^,^16^, and as antitumor therapeutics^17^,^18^. Chitosan (CH) is a particularly attractive option for clinical and biological applications because of its low immunogenicity, low toxicity, biocompatibility, and biodegradability^19^,^20^,^21^,^22^. In addition to the advantage of being positively charged because of the protonated amine groups, the efficiency of CH binding to DCs is high owing to electrostatic interactions, therefore, CH has gained wide acceptance as a drug carrier in nanomedicine^23^. These advantages motivated us to ask whether CH-NPs can increase the uptake efficiency of an adjuvant or antigen by DCs after vaccination and possibly increase antigen-specific CD8+ T cell responses *in vivo* without *ex vivo* manipulation.

Since DC-based cancer immunotherapy using NPs is advancing rapidly, NP systems have been used as a carrier to deliver an adjuvant or antigen into DCs *ex vivo*. In contrast, our system is capable of driving *in vivo* DC maturation and activation after direct injection into the body without *ex vivo* manipulation. In this study, we demonstrated a novel injection route for NPs to enhance therapeutic efficacy, whereby NPs can be taken up by DCs *in vivo*. Moreover, we demonstrated a stepwise immune response driven by DCs containing NPs.

Here, we developed a CH-NP system as a direct *in vivo* injection carrier encapsulating both OVA and poly I:C, resulting in increased efficiency of intracellular delivery of payloads to DCs, promotion of DC maturation, and activation of cytotoxic T cells via antigen-specific cross-presentation. We demonstrated that the CH-NP platform is a highly efficient delivery system that increases the uptake of an payloads by DCs *in vivo* in multiple animal tumor models, resulting in therapeutic efficacy of this direct *in vivo* injection approach without *ex vivo* manipulation of DCs.

## Results

### Characteristics of CH-NPs

**Figure 1:**
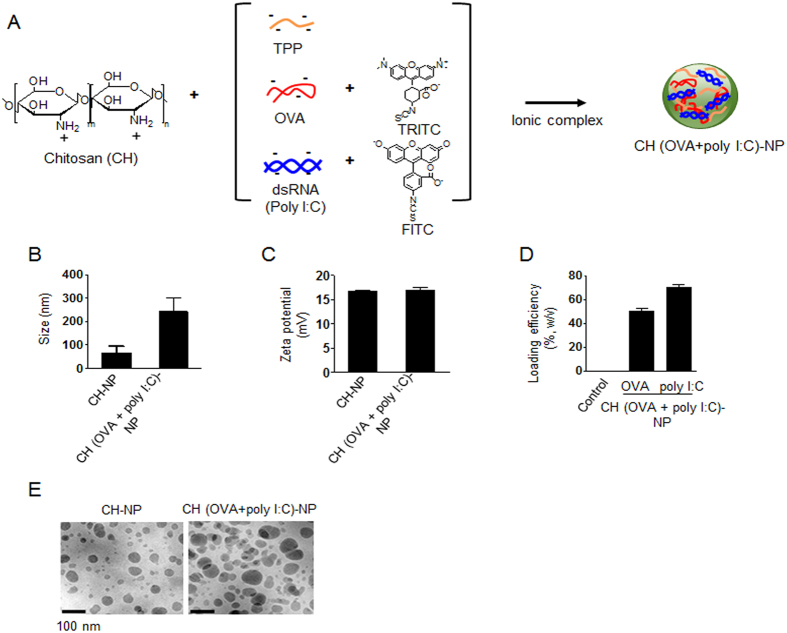
Physical properties of CH (OVA+poly I:C)-NPs. (**A**) CH (OVA+poly I:C)-NPs prepared by ionic interaction of anionic TPP, OVA, and poly I:C with cationic CH molecules. (**B**) Size and (**C**) zeta potential of the CH-NPs and CH (OVA+poly I:C)-NPs. (**D**) Individual loading efficiency of OVA and poly I:C into CH (OVA+poly I:C)-NPs. (**E**) TEM images of CH-NPs and CH (OVA+poly I:C)-NPs. Scale bar: 100 nm. Error bars represent s.e.m.

In this study, we used CH as the polymer matrix because it is particularly suitable for clinical and biological applications owing to its low toxicity, biocompatibility, biodegradability, and low immunogenicity^19^,^24^. We successfully developed and fabricated CH-NPs by ionic gelation of CH by means of anionic sodium tripolyphosphate (TPP), with encapsulation of OVA and poly I:C within the cationic CH (Fig. 1A). The structure of the CH-NP complexes was confirmed by FT-IR (Supplementary Fig. S1). First, we measured the physical properties of the CH-NPs and CH (OVA+poly I:C)-NPs. The mean particle size of CH-NPs and CH (OVA+poly I:C)-NPs was 70 ± 3.7 nm (polydispersity index, PDI: 0.258) and 254 ± 3.2 nm (PDI: 0.233), respectively (Fig. 1B). Representative histograms of their size distributions and PDI are shown in Supplementary Fig. S2. In addition, the zeta potentials of both NP types were around 15 mV (Fig. 1C). The loading efficiency of OVA and poly I:C was 50% and 70%, respectively (Fig. 1D). Additionally, the morphologies of the CH-NPs and CH (OVA+poly I:C)-NPs were examined by transmission electron microscopy (TEM). CH-NPs and CH (OVA+poly I:C)-NPs are spherical with a diameter of 50–200 nm (Fig. 1E). To confirm the release pattern of the payload, we assessed the release of OVA from CH (OVA+poly I:C)-NPs at pH4 and 37 °C, thereby mimicking the intracellular acidic environment after the uptake of CH (OVA+poly I:C)-NPs. Although the OVA release from the CH (OVA+poly I:C)-NPs at 4 °C and pH4 or pH7 was limited, it increased significantly at 37 °C and pH4 (Supplementary Fig. S3). This result indicated that drugs carried by CH (OVA+poly I:C)-NPs could be specifically released in an intracellular acidic environment.

### Intracellular delivery of CH (OVA+poly I:C)-NPs to DCs

**Figure 2:**
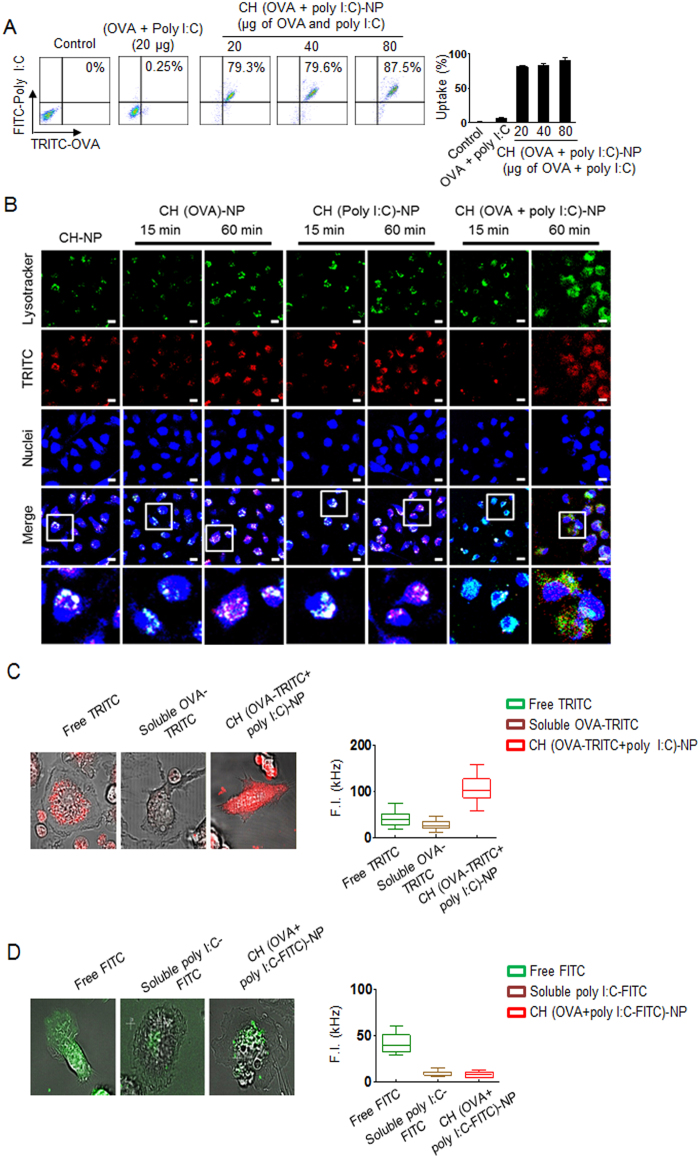
Intracellular delivery of CH (OVA+poly I:C)-NPs into DCs. (**A**) Efficiency of intracellular delivery of CH (OVA+poly I:C)-NPs into DC. OVA was labeled with TRITC, and poly I:C was labeled with FITC as indicators for visualization. (**B**) A photograph of Intracellular delivery of CH (OVA+poly I:C)-NPs into DCs. Red: TRITC-labeled OVA or TRITC-labeled poly I:C. Blue: nuclei. Scale bar: 10 μm. (**C**) Cytosolic distribution of TRITC-labeled OVA released from CH (OVA+poly I:C)-NPs after 1 hr intracellular trafficking in live DCs, and mean fluorescence intensity of the region of interest (ROI) in the cytosol of DCs. (**D**) Cytosolic distribution of FITC-labeled poly I:C released from CH (OVA+poly I:C)-NPs after 1 hr intracellular trafficking in live DCs and mean fluorescence intensity of the ROI in the cytosol of DCs. Error bars represent s.e.m.

We next assessed the intracellular delivery of CH-NPs by flow cytometry and confocal microscopy (Fig. 2A and B). Prior to this assay, we conjugated tetramethylrhodamine (TRICT) with OVA and fluorescein isothiocyanate (FITC) with poly I:C as fluorescent indicators to confirm the intracellular delivery and trafficking of OVA or poly I:C in DCs. Flow cytometric analysis revealed that the CH (OVA+poly I:C)-NPs underwent highly efficient intracellular uptake as compared to control DCs (Fig. 2A). In addition, confocal microscopic analysis showed that the uptake of CH (OVA+poly I:C)-NPs by DCs was consistent with the flow cytometric data (Fig. 2B). We also confirmed uptake of CH (OVA+poly I:C)-NPs by DCs at different temperatures (Supplementary Fig. S4). Moreover, we verified the trafficking of OVA and poly I:C (separately) released from CH (OVA+poly I:C)-NPs into DCs at the single cell level using confocal microscopy (Fig. 2C and D). Although soluble OVA or poly I:C can barely penetrate DCs, OVA (red color) from CH (OVA+poly I:C)-NPs showed more homogeneous distribution throughout a DC, specifically in the cytosol and endosomes (Fig. 2C), and poly I:C was effectively localized to endosomes in a DC (Fig. 2D). For this reason, OVA from CH (OVA+poly I:C)-NPs was observed at some distance from the lysosomes, suggesting that OVA was released from the endosomes. In contrast, poly I:C was localized to endosomes. Additionally, we measured DC viability after incubation with the CH-NPs to evaluate cytotoxicity. The results showed that CH-NPs are not toxic to DCs (Supplementary Fig. S5).

### *In vitro* DC maturation and activation under the influence of CH (OVA+poly I:C)-NPs

**Figure 3:**
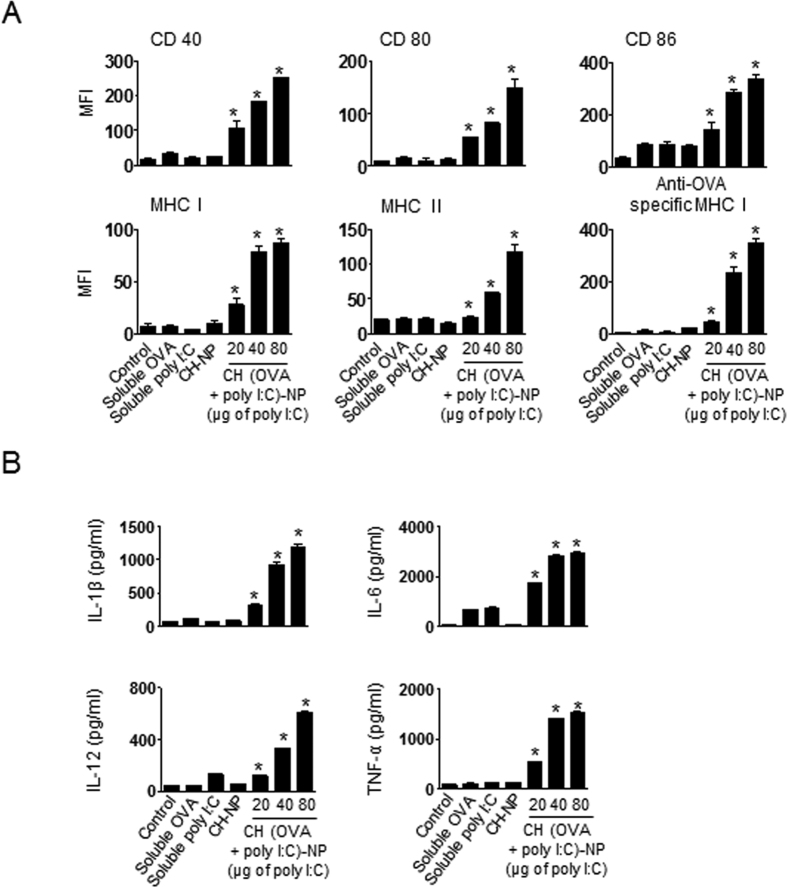
Maturation and activation of DCs induced by CH (OVA+poly I:C)-NPs. (**A**) Surface maturation markers (CD40, CD80, CD86, MHC class I, MHC class II, and OVA-specific MHC class I) were confirmed by flow cytometric analysis. (**B**) ELISA quantification of pro-inflammatory cytokines and DC activation factors in culture supernatants of DCs incubated with CH (OVA+poly I:C)-NPs for 24 h. Error bars represent s.e.m. *p < 0.05.

To assess the *in vitro* maturation and activation of DCs, we characterized the DCs in terms of the expression of activation markers and pro-inflammatory cytokines^25^. DCs were isolated from the bone marrow of C57BL/6 mice and incubated with CH (OVA+poly I:C)-NPs (20 μg, 40 μg, or 80 μg in terms of poly I:C). DCs treated with CH (OVA + poly I:C)-NPs showed significantly higher expression of surface maturation markers such as CD40, CD80, CD86, MHC class I, and MHC class II as compared to untreated DCs, DCs treated with soluble OVA, DCs treated with soluble poly I:C, and DCs treated with CH-NPs without OVA and poly I:C (Fig. 3A). These results indicated that CH (OVA + poly I:C)-NPs promoted DC maturation and activation. Notably, OVA-specific MHC class I expression of the DCs treated with CH (OVA+poly I:C)-NPs was significantly higher than that of untreated DCs and DCs treated with CH-NPs (Fig. 3A). In addition, DCs treated with CH (OVA + poly I:C)-NPs showed a significant increase in the expression of pro-inflammatory cytokines IL-1β, IL-6, IL-12p70, and TNF-α during DC maturation in comparison with untreated DCs and other DC treatment groups (Fig. 3B). Collectively, these data revealed that CH (OVA+poly I:C)-NPs induced maturation, activation, and antigen-specific MHC class I expression in DCs.

### An *in vivo* immune response after CH (OVA+poly I:C)-NP injection

**Figure 4:**
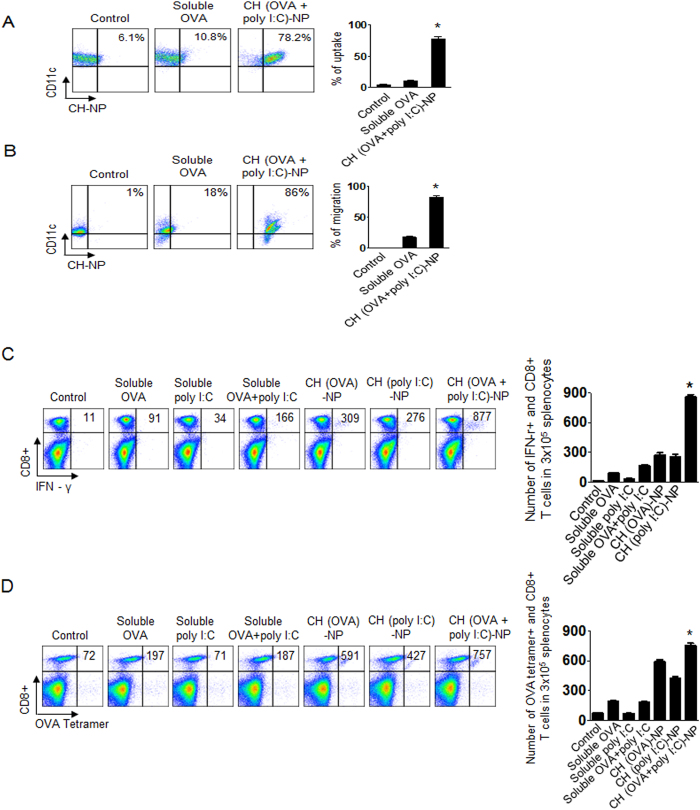
An *in vivo* stepwise CH (OVA+poly I:C)-NP-based active immune response. (**A**) CH (OVA+poly I:C)-NP uptake by DCs after i.p. injection (*p < 0.001). The uptake was analyzed by flow cytometry. CH (OVA+poly I:C)-NPs were labeled with TRITC (red), and DCs were stained with an anti-CD11c antibody. (**B**) Migration of DCs containing CH (OVA+poly I:C)-NPs to the intraperitoneal lymph node. Mouse intraperitoneal lymph nodes were collected and analyzed by flow cytometry for the DCs (stained with anti-CD11c antibody) and CH (OVA+poly I:C)-NPs labeled with TRITC (*p < 0.001). (**C**) Cytotoxic CD8+ T cell activation was assessed in the splenocytes of the immunized mice by flow cytometric analysis for cells positively stained with anti-CD8 and anti-IFN-γ antibodies. The bar graph depicts the number of CD8+ T cells among the splenocytes (*p < 0.001). (**D**) The OVA-specific CD8+ T cells among total CD8 T cells according to flow cytometric analysis for cells positively stained with anti-CD8 and anti-OVA tetramer antibodies. The bar graph depicts the number of antigen-specific CD8+ T cells (*p < 0.001). Error bars represent s.e.m.

To assess the *in vivo* stepwise immune response based on the CH-NP platform in C57BL/6 mice, we considered the following mechanism of the immune response: (1) uptake of NPs by naive DCs after intraperitoneal (i.p.) injection, (2) migration of DCs containing CH-NP to a site near lymph nodes, and (3) activation of antigen-specific cytotoxic CD8+ T cells by mature DCs. Therefore, we first assessed the *in vivo* uptake of CH (OVA+poly I:C)-NPs by DCs after i.p. injection into mice by flow cytometric analysis for CD11c+ and TRITC-labeled OVA+ cells. Uptake of CH (OVA+poly I:C)-NPs by DCs was significantly increased compared to soluble OVA (*p < 0.001, Fig. 4A). We next evaluated the migration of DCs containing CH (OVA+poly I:C)-NP to an intraperitoneal lymph node and spleen by flow cytometry. DCs containing CH (OVA+poly I:C)-NPs significantly migrated to the lymph nodes (*p < 0.001, Fig. 4B) and spleen (*p < 0.001, Supplementary Fig. S6) compared to soluble OVA. We next assessed antigen-specific activation of cytotoxic CD8+ T cells by mature DCs containing CH (OVA+poly I:C)-NPs by flow cytometric analysis for anti-CD8 and anti-IFN-γ staining. The number of IFN-γ secreting cytotoxic CD8+ T cells significantly increased in the CH (OVA+poly I:C)-NPs injected mice compared to the other treatments (*p < 0.001, Fig. 4C). In addition, even if we injected CH (OVA+poly I:C) into the mice subcutaneously (s.c.), the number of IFN-γ secreting cytotoxic CD8+ T cells significantly increased (*p < 0.001, Supplementary Fig. S7). To confirm the antigen-specific CD8+ T cells among all CD8+ T cells, we finally quantified OVA-specific CD8+ T cells by anti-OVA tetramer staining^26^. The number of OVA-specific CD8+ T cells was significantly increased in the CH (OVA+poly I:C)-NP injected mice as compared to the other treatment groups (*p < 0.001, Fig. 4D). These results indicated an active immune response after CH (OVA+poly I:C)-NP injection into mice without *ex vivo* manipulation.

### Therapeutic efficacy of CH (OVA+poly I:C)-NPs

**Figure 5:**
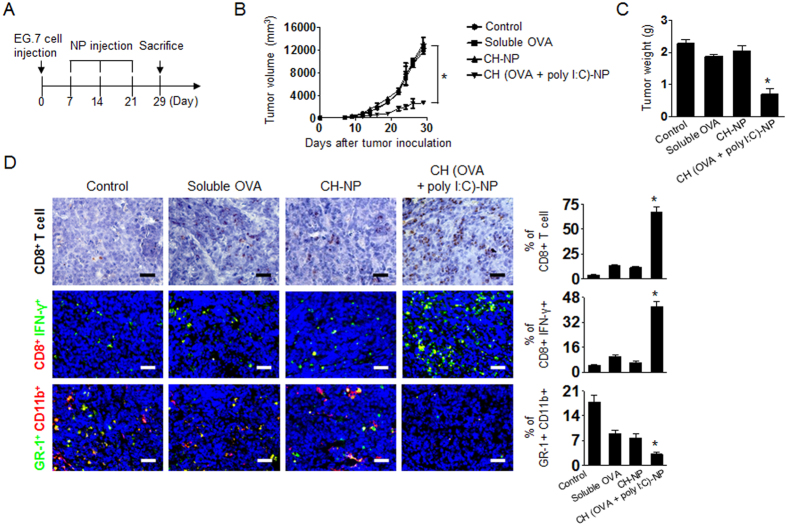
Antitumor efficacy of CH (OVA+poly I:C)-NP treatment in the EG.7 tumor model. Treatment began 1 week after s.c. injection of tumor cells into the mice. Control, soluble OVA, CH-NPs, or CH (OVA+poly I:C)-NPs were injected three times at weekly intervals at a dose of 100 μg of OVA and 80 μg of poly I:C via i.p. injection. (**A**) The schedule of the CH (OVA+poly I:C)-NP-based antitumor treatment. (**B**) Tumor volume (*p < 0.001) and (**C**) tumor weight (*p < 0.001) after treatment with the various formulations. (**D**) IHC analysis of CD8+ T cell localization in tumor tissue (anti-CD8 staining, scale bar: 50 μm) was performed on EG.7-tumor tissues. The bar graph indicated % of CD8+ T cells (brown color)/total tumor cells (blue color) in same tissue area. Immunofluorescence staining for cytotoxic CD8+ T cells (positive for anti-CD8 and IFN-γ immunostaining) was also performed. The bar graph indicated % of CD8+IFN-γ+ cells (CD8: red color, IFN-γ: green color)/total tumor cells (blue color) in same tissue area. The MDSC population in tumor tissue was confirmed by staining with anti-GR-1 and anti-CD11b antibodies. The bar graph indicated % of GR-1+CD11b+ cells (GR-1: green color, CD11b: red color)/total tumor cells (blue color) in same tissue area. Scale bar: 100 μm. All analyses were performed in five random fields recorded for each slide. Error bars represent s.e.m. *p < 0.001.

To determine the potential therapeutic efficacy of CH (OVA+poly I:C)-NPs, we selected EG.7-OVA cells, which are attractive tumor cells for studying an OVA-based mouse model in terms of immunotherapy^27^,^28^. Seven days after the s.c. injection of EG.7-OVA tumor cells into C57BL/6 mice, the mice were randomly allocated to the following groups (n = 6 mice per group): (1) control, (2) soluble OVA (100 μg), (3) CH-NPs without OVA and poly I:C, and (4) CH (OVA+poly I:C)-NPs (100 μg of OVA and 80 μg of poly I:C). The experimental groups underwent three i.p. injections at weekly intervals 7 days after tumor inoculation (Fig. 5A). CH (OVA+poly I:C)-NP injected mice showed significantly higher inhibition of tumor growth as compared to the control group, soluble OVA, or CH-NP injected mice (*p < 0.001, Fig. 5B). Notably, the tumor weight in the CH (OVA+poly I:C)-NP injected mice was significantly lower than that of the control group (67% reduction; p < 0.02), soluble OVA (56% reduction; p < 0.01), and CH-NP injected mice (60% reduction; p < 0.03, Fig. 5C). We also confirmed therapeutic efficacy of CH (OVA)-NPs and CH (poly I:C)-NPs. Although CH (OVA)-NPs and CH (poly I:C)-NPs showed therapeutic efficacy, CH (OVA+poly IC)-NPs showed stronger inhibition of tumor growth (Supplementary Fig. S8). There were no differences in the total body weight, feeding habits, or behavior between the groups, suggesting that there were no overt adverse effects related to the treatment.

To determine the possible mechanisms underlying the efficacy of CH (OVA+poly I:C)-NP treatment in tumor tissues, we examined the tumors for markers of CD8+ T cells (anti-CD8 immunostaining), cytotoxic CD8+ T cells (anti-CD8 and anti-IFN-γ immunostaining), and myeloid-derived suppressor cells (MDSCs, anti-GR-1 and anti-CD11b immunostaining, Fig. 5D). In the immunohistochemical (IHC) assay, the CH (OVA+poly I:C)-NP-treated group showed a significantly greater population of CD8+ T cells in tumor tissue as compared to the other groups (*p < 0.001). In the immunofluorescence assay, the CH (OVA+poly I:C)-NP-treated group showed a significantly greater population of CD8/IFN-γ expressing cytotoxic CD8+ T cells (*p < 0.001), and a decreased population of MDSCs (GR-1+ and anti-CD11b+) as compared to the control or the other treatment groups (*p < 0.001, Fig. 5D).

In addition, we confirmed therapeutic efficacy of CH (OVA+poly I:C)-NPs in a TC-1 tumor model (OVA-negative tumor) as an irrelevant antigen model (Supplementary Fig. S9). Additionally, we confirmed the therapeutic efficacy of CH (OVA+poly I:C)-NPs at a different number of injection time points (Supplementary Fig. S10). In the TC-1 tumor model, CH (OVA+poly I:C)-NPs showed no therapeutic effect as compared to the control (p < 0.03, Supplementary Fig. S9). Moreover, two-time injection caused stronger inhibition of tumor growth compared to a single injection. This finding may be attributed to a CD8+ T cell busting effect for vaccination (Supplementary Fig. S10).

**Figure 6:**
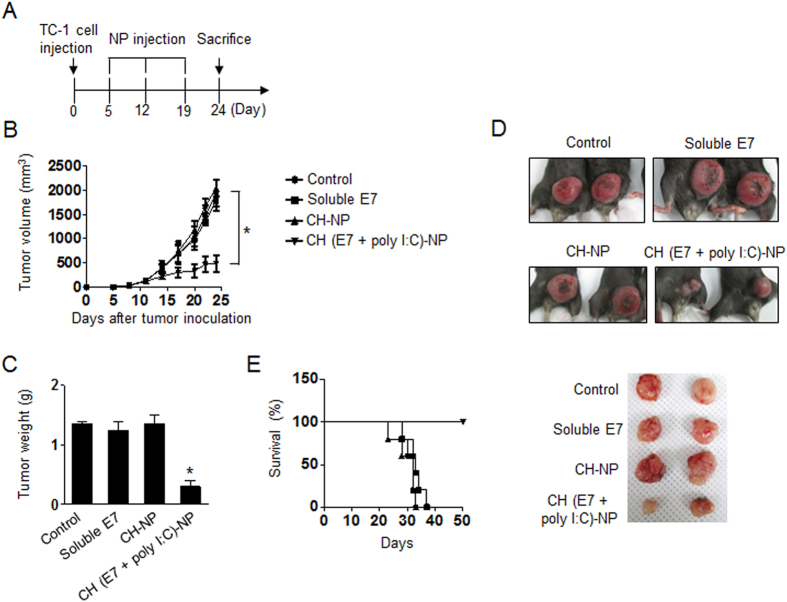
Antitumor efficacy of CH (E7+poly I:C)-NPs in the TC-1 tumor model. Treatment began 1 week after s.c. injection of tumor cells into mice. Control, soluble E7, CH-NPs, or CH (E7+poly I:C)-NPs were injected thrice weekly at a dose of 100 μg of E7 and 80 μg of poly I:C via i.p. injection. (**A**) The schedule of the CH (E7+poly I:C)-NP-based antitumor treatment. (**B**) Tumor volume (*p < 0.001) and (**C**) tumor weight (*p < 0.001) after treatment with the various formulations. (**D**) Representative images of the treated mice. (**E**) The survival curve of mice. Error bars represent s.e.m.

To ensure that the effects of CH-NPs are not unique to just one target antigen, we performed an *in vivo* experiment with additional target antigens. We prepared E7 peptide encapsulating CH (E7+poly I:C)-NPs and used them against the TC-1 tumor model, where tumor cells express HPV16 and HPV-E7 proteins^29^,^30^. The physical properties of the CH (E7+poly I:C)-NPs are shown in Supplementary Fig. S11. Mice were randomly allocated to the following groups (n = 6 mice per group): (1) control, (2) soluble E7 (100 μg), (3) CH-NPs, and (4) CH (E7+poly I:C)-NPs (100 μg of E7 peptide and 80 μg of poly I:C). The experimental groups received three i.p. injections at weekly intervals 7 days after tumor inoculation (Fig. 6A). Treatment with CH (E7+poly I:C)-NPs resulted in significant inhibition of tumor growth and weight as compared to the control (78% reduction; p < 0.01), soluble E7 (76% reduction; p < 0.03), and CH-NP (78% reduction; p < 0.02, Fig. 6B,C and D). Notably, 100% of mice vaccinated with CH (E7+poly I:C)-NPs survived for at least 50 days, while mice vaccinated with control, soluble E7, or CH-NP died within 40 days after tumor inoculation (Fig. 6E). These data suggested that vaccination with CH (E7+poly I:C)-NPs can enhance therapeutic antitumor efficacy of E7 peptide and prolong survival of vaccinated mice.

## Discussion

We demonstrate here that a novel *in vivo* active cancer immunotherapy based on CH-NP loaded with an adjuvant and antigen to increase *in vivo* maturation of DCs leads to potent antigen-specific CD8+ T cell immunity after direct injection of such CH-NPs into tumor-bearing mice. This approach has broad applicability to active delivery of adjuvants and antigens to DCs without *ex vivo* manipulation, leading to strong cytotoxic CD8+ T cell-mediated immune responses and increased therapeutic efficacy. Moreover, CH-NPs effectively increase immune responses to multiple antigen-specific targets of interest thereby enhancing therapeutic efficacy.

Cancer immunotherapy is an active area of cancer research. Many scientists worldwide are exploring advanced treatment methods to develop targeted immunotherapies such as monoclonal antibodies or cancer vaccines^1^,^4^. Although these studies have resulted in improvements of therapeutic effectiveness, most have not been as successful as expected. Therefore, the development of novel systems and elucidation of their mechanisms of action would be worthwhile. The most common method for immunotherapy is delivery of adjuvants and antigens to DCs to initiate their maturation, which is a key parameter for medical and pharmaceutical applications of cancer immunotherapy^5^,^7^. Nonetheless, the medical, economic, and logistic complexities associated with *ex vivo* manipulation of DCs are substantial and need to be overcome to realize the full potential of DCs in clinical settings^31^. To overcome these limitations, direct injection systems need to be developed with technologies that effect robust and simple delivery as seen in nanomaterial carrier systems without *ex vivo* manipulation.

Here, we demonstrate a novel therapeutic strategy for DC-based cancer immunotherapy without *ex vivo* manipulation of DCs. Our system involves direct injection of custom-designed NPs into tumor-bearing mice to target DCs. Therefore, we can decrease contamination and improve cost effectiveness and safety of DC-based cancer immunotherapy. In the field of DC-based cancer immunotherapy, NP system has been used as an effective antigen or adjuvant delivery carrier to increase the antigen’s or adjuvant’s efficiency of penetration into DCs through *ex vivo* manipulation^3^,^32^,^33^. Moreover, NP system has also been utilized for delivery of an antigen or adjuvant cargo by intratumoral injection to increase the immune response mediated by DCs in the tumor microenvironment^34^,^35^. However, exact injection into a tumor nodule is difficult because of organ specific peculiarities of growth of tumor nodules (small size and/or deep location in some cases). These problems are a hurdle for NP-based cancer immunotherapy. Therefore, we expand that if NPs can be injected directly into tumor-bearing mice without *ex vivo* manipulation, then DC-based immunomodulation will be dramatically more effective without the above limitations. In addition, we demonstrate *in vivo* therapeutic efficacy in two tumor models.

A number of NP systems have been tested for therapeutic application because of NPs’ low toxicity, low immunogenicity, and suitable systemic distribution in the body. These systems include microparticles, nanofibres, metal-based particles, and emulsions. Although many compounds are potentially useful as delivery agents, some of these have problems with safety and evoked immune responses. The development of active vaccination strategies involving NP systems for immunotherapy therefore requires clinically suitable, safe, and effective delivery. NPs are a promising and desirable carrier because of their potential to overcome the limitations of DC or T cell-based immunotherapy. Moreover, packaging of therapeutic payloads into NPs may be a clinically viable approach to the development of vaccine-related immunotherapies.

CH-NPs are an attractive NP platform for payload delivery because of their biocompatibility, biodegradability, low toxicity, and low immunogenicity, which are key parameters for medical and pharmaceutical applications^24^,^36^. Moreover, CH-based drug delivery systems are widely used for cancer chemotherapy and other treatments. They have been designed to increase or facilitate uptake into target tissues, protect payloads, and reduce nonspecific delivery. Furthermore, NP-transported payloads are frequently located within the particles, and therefore this incorporation of adjuvants or antigens may increase efficiency of the uptake into target cells such as DCs. Here, we developed CH-NPs as a systemic delivery carrier encapsulating OVA and poly I:C for i.p. and s.q. injection into tumor-bearing mice. These NPs can be taken up by DCs *in vivo*, leading to activation of cytotoxic CD8+ T cell immunity.

NP-based cancer immunotherapy offers larger payloads of antigens or adjuvants than antibodies do, as evidenced by the potent immune response elicited in mice in the present study. In addition, the NP system allows for co-delivery of therapeutic payloads, such as antigen (protein or peptide) or adjuvant that may further enhance the antigen-specific immune response without increasing toxicity. This NP-based delivery system may be attractive for diverse biomedical applications. Although the CH-NP platform may be effective against diseases associated with the immune system and for enhancement of immune responses, additional possibilities such as optimized loading for effective cytokine or immune modulation can be explored and advanced systems can be developed for research purposes.

In addition, NP-based active cancer immunotherapy has the benefit of reduced undesirable *ex vivo* manipulations for the production of cytotoxic CD8+ T cells that can kill tumor cells. Therefore, NP-based immunotherapies make it possible to achieve robust immune responses without adverse effects of matrix toxicity at the site of administration. Our data show that CH-NP-based direct adjuvant and antigen delivery without *ex vivo* manipulation can invoke antigen-specific CD8+ T cell immunity. This CH-NP-based platform can be used for delivery of many other target adjuvants or antigens. In addition, the CH-NP system can be expanded and developed to include additional therapeutic and experimental approaches. The CH-NP-based strategy presented here has broad applicability as a delivery platform for enhancement of immune responses in active cancer immunotherapy by direct injection into tumor-bearing mice and may be adapted to other immunological diseases.

## Methods

### Materials

CH (MW 50–190 KDa), TPP, OVA, and poly I:C were purchased from Sigma-Aldrich (St. Louis, MO, USA). FITC-conjugated anti-mouse IFN-γ, PE-conjugated anti-mouse CD40, and anti-OVA-specific (SIINFEKL/H-2Kb) antibodies and mouse TNF-α, IL-1β, IL-6, and IL-12p70 ELISA Ready-SET-Go kits were purchased from eBioscience (San Diego, CA, USA). A FITC-conjugated anti-mouse CD11c antibody and PE-conjugated anti-mouse CD8a, CD80, CD86, MHC class I, and MHC class II antibodies and a mouse IFN-γ ELISA kit were purchased from Biolegend (San Diego, CA, USA). RPMI 1640 and fetal bovine serum (FBS) were acquired from Biowest (Nuaille, France). Granulocyte-macrophage colony-stimulating factor (GM-CSF) was purchased from JW Creagene (Gyeonggi, South Korea). HPV-16 E7 peptide (MW 2.4 kDa, AGQAEPDRAHYNIVTFCCKCDS)^37^ was purchased from AnyGen Co. (Seoul, South Korea). All other materials were of analytical grade and used without further purification.

### Preparation of CH-NPs

CH-NPs were prepared by ionic gelation of CH by means of anionic TPP with encapsulation of OVA and poly I:C. Briefly, 180 μL of TPP (0.25% w/v), 250 μL of OVA (1 mg/mL), and 10 μL of poly I:C (10 mg/mL) were added to 1 mL of a CH solution (2 mg/mL), and CH-NPs spontaneously formed with constant stirring at room temperature. After incubation at 4 °C for 30 min, CH-NPs were collected by centrifugation at 15,814 × *g* for 50 min at 4 °C. The pellet was washed thrice with sterile water and the isolated CH-NPs were stored at 4 °C until use. Size and zeta potential of CH-NPs were measured by light scattering using a particle size analyzer and Zeta Plus (Brookhaven Instrument Co., CA, USA), respectively. Loading efficiency of OVA or poly I:C was measured by the BCA assay method^27^ or NanoDrop^38^ (ND-1000 spectrophotometer, NanoDrop Technology, USA), respectively, at a wavelength of 260 nm. After centrifugation of CH (OVA+poly I:C)-NPs, we collected supernatant and measured the concentration of OVA and poly I:C. The OVA or poly I:C loading efficiency was calculated as follows^39^; loading efficiency (%) = [(*F*_i_-*F*_t_)/*F*_i_] × 100. Where *F*_t_ is the concentration of OVA or poly I:C in the supernatant and *F*_i_ is the initial concentration of OVA or poly I:C. Morphological characteristics of CH-NPs were examined under a field emission transmission electron microscope (FETEM, JEOL, 200 kV, USA)^40^. To assess the release of OVA from CH-NPs in an acidic medium mimicking intracellular environment, we measured OVA concentration by the BCA assay after CH-NPs were diluted in a 0.9% NaCl solution with pH adjusted by means of 0.1 M HCl to pH 4 and were incubated at 4 °C or 37 °C for a predetermined period, and poly I:C was quantified by means of the NanoDrop.

### Mice and cell lines

Female C57BL/6 mice (5–6 weeks old, 20 g) were purchased from ORIENT (Gapyeong, South Korea) and maintained according to the protocols approved by the Konkuk University Institutional Animal Care and Use Committee (Ref. No.: KU14157). All the procedures were performed according to approved protocols and in accordance with recommendations for the proper use and care of animals at the specific pathogen-free housing facility at Konkuk University. OVA expressing EG.7 lymphoma cells (EL4 cell line transfected with the gene encoding OVA) and TC-1 cells expressing HPV16 and HPV-E7 proteins were cultured in the RPMI 1640 medium supplemented with 10% FBS and 0.1% gentamycin.

### Generation of DCs from mouse bone marrow

DCs were harvested from the bone marrow of C57BL/6 mice^41^. Briefly, bone marrow was collected from the tibiae and femora of the mice. Red blood cells were depleted using RBC-lysis buffer (Sigma-Aldrich), and bone marrow cells (2 × 10^6^ cells/well) were collected and cultured in a 6-well plate containing 4 mL of the RPMI1640 supplemented with 10% of FBS, 0.1% of gentamycin, and 20 ng/mL mouse recombinant GM-CSF at 37 °C in a 5% CO_2_ incubator. The DCs were used after 6 days of culture.

### Intracellular delivery of CH (OVA+poly I:C)-NPs in DCs and trafficking assay

Prior to testing of intracellular delivery of CH-NPs, we conjugated the fluorescent dye TRITC with OVA and FITC with poly I:C for flow cytometric and confocal microscopic analyses, respectively. Briefly, DCs were incubated with CH (OVA+poly I:C)-NPs for 15 min or 60 min at room temperature. After that, CH (OVA+poly I:C)-NP uptake by DCs was analyzed by flow cytometry (FACSCalibur with CellQuest software, BD Biosciences, Franklin Lakes, NJ, USA) and confocal microscopy (DeltaVisionTM PD, GE Healthcare, Piscataway, NJ, USA). To confirm intracellular delivery of CH (OVA)-NPs, CH (poly I:C)-NPs, and CH (OVA+poly I:C)-NPs, we incubated DCs with CH-NPs for 15 min. After that, DCs were fixed in a 4% paraformaldehyde solution for 10 min, and then stained with 1 μM Lysotracker Green (Cell Signaling Technology) and 1 μM To-pro-3 (blue, Thermo Fisher Scientific) for 30 min. The samples were examined by confocal microscopy. Fluorescence imaging of live DCs was performed using an inverted confocal microscope (LSM 780, Carl Zeiss, Jena, Germany). TRITC was excited at 561 nm through a water immersed objective lens (C-Apochromat, 40×, 1.2NA; Carl Zeiss), and the fluorescent signal was detected in the range of 575 nm to 670 nm in photon counting mode. The pinhole diameters for confocal imaging were adjusted to 34 μm (1 Airy unit). To quantify mean fluorescence intensity, a circular region of interest (ROI) was positioned in the cytosol of DCs (n = 10) at specific distances from a perinuclear region that contained many vesicles such as endosomes and lysosomes. Bright aggregates at the membrane and in the perinuclear region were excluded from the ROI to evaluate only the release of OVA or poly I:C into the cytosol^42^.

### *In vitro* assessment of DC maturation and cytokine secretion

To evaluate DC maturation, DCs were cultured in 6-well plates at the density of 2 × 10^6^ cells per well and allowed to adhere overnight. DCs alone as a control, soluble OVA (100 μg), soluble poly I:C (80 μg), CH-NPs, or CH (OVA+poly I:C)-NPs (OVA 100 μg + poly I:C 80 μg) were incubated for 30 min, and then the CH-NP containing medium was removed. DCs were incubated for additional 24 h, then DC maturation was analyzed by flow cytometry. DCs were stained with FITC-conjugated anti-CD11c and PE-conjugated anti-CD40, anti-CD80, anti-CD86, anti-MHC class I, anti-MHC class II, and anti-OVA-specific (SIINFEKL/H-2Kb) MHC class I antibodies. In addition, cytokines (IL-1β, IL-6, IL-12p70, and TNF-α) secreted from DCs during maturation were analyzed by means of cytokine-specific ELISA kits (eBioscience, USA).

### *In vivo* DC uptake, migration, and T cell activation

To quantify *in vivo* CH-NP uptake by DCs, we first assessed DC uptake by flow cytometry. We conjugated OVA with TRITC for flow cytometric analysis. We injected PBS as a control, soluble OVA (100 μg), or CH (OVA + poly I:C)-NPs via the i.p. route in mice (n = 5 mice per group). After 2 h, we collected ascites from the mice, and centrifuged them at 158 × *g* for 5 min to collect the cells. The latter were stained with a FITC-labeled anti-CD11c antibody, then analyzed by flow cytometry to quantify the TRITC-OVA+/FITC-CD11c+ DCs. We next assessed migration of DCs containing CH (OVA+poly I:C)-NPs. We injected PBS as a control, soluble OVA (100 μg), or CH (OVA + poly I:C)-NPs via the i.p. route into mice (n = 5 mice per group). After 36 h, the mice were euthanized, and the intraperitoneal lymph nodes were collected. The lymph nodes were strained and the cells were collected by centrifugation at 158 × *g* for 5 min. We then stained the cells with the FITC-labeled anti-CD11c antibody and analyzed them by flow cytometry to confirm whether DCs containing CH (OVA+poly I:C)-NPs had migrated to the lymph nodes. Finally, we assessed activation of cytotoxic CD8+ T cells by means of DCs matured by treatment with CH (OVA+poly I:C)-NP. We injected PBS as a control, soluble OVA, soluble poly I:C, soluble OVA+poly I:C, CH (OVA)-NPs, CH (poly I:C)-NPs, or CH (OVA + poly I:C)-NPs via the i.p. route into mice (n = 5 mice per group). Seven days after the last vaccination (three injections at weekly intervals), we euthanized the mice and collected their spleens. Splenocytes (1 × 10^7^) harvested from the vaccinated mice were resuspended in 1 mL of RPMI 1640 with 10% of FBS, 0.1% of gentamycin, and 0.5% of β-mercaptoethanol, and incubated for 16 h with GolgiPlug (BD Biosciences) and the OVA peptide (1 μg/mL). The cells were washed and stained with a PE-conjugated anti-CD8a antibody and a FITC-conjugated anti-IFN-γ antibody to confirm activation of IFN-γ secreting CD8+ T cells. Additionally, we confirmed the antigen-specific CD8 T cells in total CD8 T cells. The cells were stained with a PE-conjugated anti-CD8a antibody and a FITC-conjugated anti-OVA tetramer antibody^26^. Data were acquired on a FACSCalibur with CellQuest software.

### Antitumor efficacy of CH (OVA+poly I:C)-NPs

To generate tumors, EG.7 cells (1 × 10^6^ cells per 0.1 mL HBSS) were injected subcutaneously (s.c.) into C57BL/6 mice. The mice (n = 6 per group) were monitored daily for adverse effects of treatment and were euthanized when the control group seemed moribund. To assess the effect on tumor growth, treatment was started 1 week after s.c. injection of the tumor cells into the mice. PBS as a negative control, soluble OVA, CH-NPs, or CH (OVA+poly I:C)-NPs were administered once a week for 3 weeks by i.p. injection. The tumor volume and tumor weight in the mice were recorded. The tumor volume was measured using calipers, and the volume was calculated using the following formula^27^: tumor volume (mm^3^) = length × (width)^2^/2. The investigators who performed the necropsies, tumor collection, and tissue processing were blinded to the treatment group assignments. Tissue specimens were fixed with either 4% paraformaldehyde or the optimum cutting temperature (OCT) compound (Miles, Inc., Elkhart, IN). For the TC-1 tumor model, TC-1 cells (1 × 10^6^ cells per 0.1 mL HBSS) were injected s.c. into the mice (n = 6 per group). To assess tumor growth, treatment was started 1 week after s.c. injection of tumor cells into the mice. PBS as a negative control, soluble E7, CH-NPs, and CH (E7+poly I:C)-NPs were administered once a week for 3 weeks by i.p. injection. Tumor volumes, tumor weights, and survival periods of the mice were recorded.

### Immunohistochemical (IHC) staining

This analysis was performed on tumor tissues from mice injected i.p. with CH (OVA+poly I:C)-NPs. IHC analyses of the CD8+ T cell population (CD8+ according to immunostaining), cytotoxic CD8+ T cells (CD8+ and IFN-γ+ according to immunostaining), OVA specific CD8+ T cells (CD8+ and OVA+ according to immunostaining), and MDSCs (GR-1+ and CD11B+ according to immunostaining) were performed as described previously^24^. All these analyses were conducted in five random fields of each slide at ×200 magnification. All staining was scored by two investigators blinded to the group assignments of mice.

### Statistical analysis

Differences in continuous variables were analyzed by Student’s *t* test for comparison of two groups, and ANOVA was performed to assess differences in multiple group comparisons. For values that were not normally distributed, the Mann-Whitney rank sum test was carried out. The Statistical Package for the Social Sciences (SPSS, Inc.) was used for all calculations. Differences with a p value of < 0.05 were considered statistically significant.

## Additional Information

**How to cite this article**: Han, H. D. *et al*. *In vivo* stepwise immunomodulation using chitosan nanoparticles as a platform nanotechnology for cancer immunotherapy. *Sci. Rep.* **6**, 38348; doi: 10.1038/srep38348 (2016).

**Publisher's note:** Springer Nature remains neutral with regard to jurisdictional claims in published maps and institutional affiliations.

## Supplementary Material

Supplementary Figures
