# Supplementary material for: In vivo stepwise immunomodulation using chitosan nanoparticles as a platform nanotechnology for cancer immunotherapy
Source: Sci Rep. 2016 Dec 2;6:38348. doi: 10.1038/srep38348 (PMC5133713; doi:10.1038/srep38348)
Supplement: Supplementary Figures [file srep38348-s1.pdf]

Supplementary Information

In vivo stepwise immunomodulation using chitosan nanoparticles as a platform nanotechnology for cancer immunotherapy

Hee Dong Han, Yeongseon Byeon, Jong-Hwa Jang, Hat Nim Jeon, Ga Hee Kim, Min Gi Kim, Chan-Gi Pack, Tae Heung Kang, In Duk Jung, Yong Taik Lim, Young Joo Lee, Jeong-Won Lee, Byung Cheol Shin, Hyung Jun Ahn, Anil K. Sood, and Yeong-Min Park

Corresponding authors:

Hee Dong Han, Department of Immunology, School of Medicine, Konkuk University, Chungju 380-701, South Korea, Phone: 82-2-2049-6273, Fax: 82-2-2049-6192, E-mail: hanhd@kku.ac.kr

Yeong Min Park, Department of Immunology, School of Medicine, Konkuk University, Chungju 380-701, South Korea, Phone: 82-2-2049-6273, Fax: 82-2-2049-6192, E-mail: immun3023@kku.ac.kr

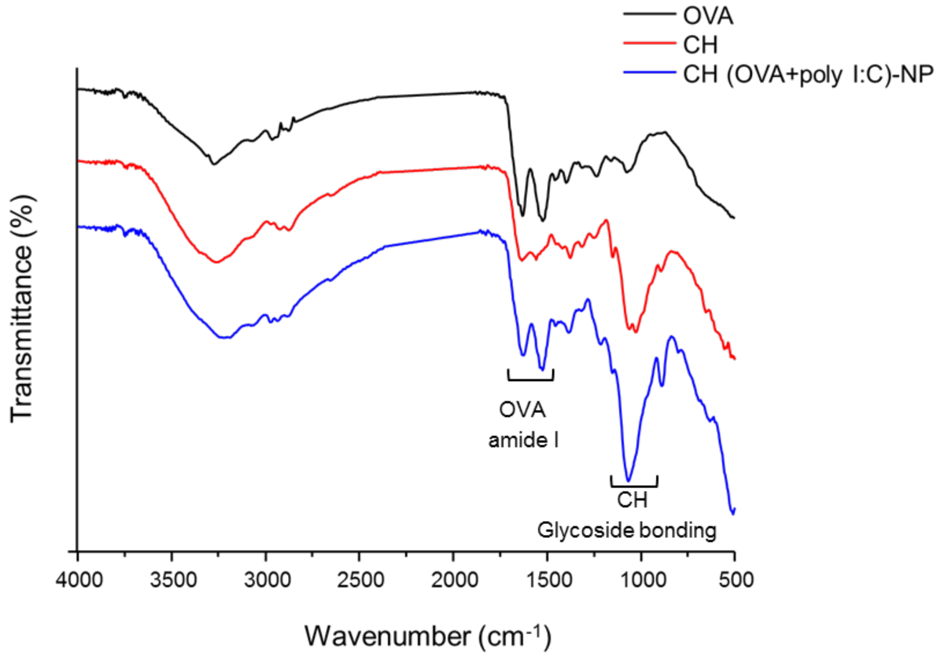

Figure S1. FT-IR spectra of CH-NP. The complex of CH (OVA+poly I:C)-NP was confirmed by CH peck (glycoside group: 1151 cm^{-1} and 888 cm^{-1}) of chitosan (CH) and amide I peck (carboxyl C=O stretch, 1520-1623 cm^{-1}) of OVA.

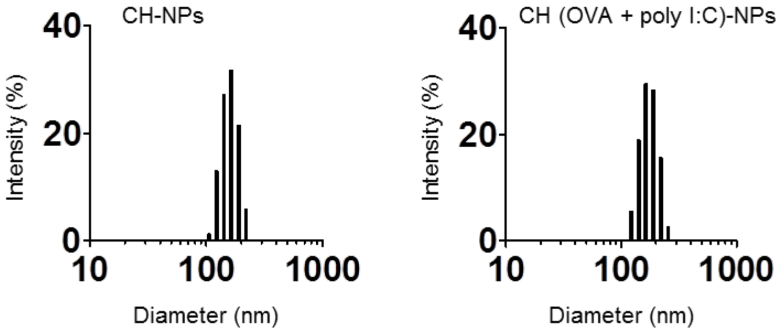

	Polydispersity index (PDI)
CH-NP	0.258
CH (OVA+poly I:C)-NP	0.233

Figure S2. Representative histograms of size distributions for CH-NPs and CH (OVA+poly I:C)-NPs.

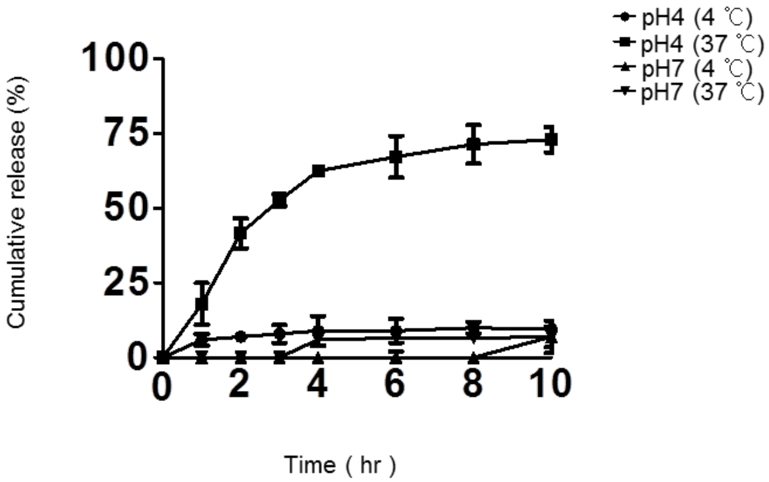

Figure S3. Release of OVA from CH (OVA+poly I:C)-NPs at 4°C or 37°C under acidic conditions that mimic the intracellular conditions. The data are represented as a mean ± S.D. (n=3).

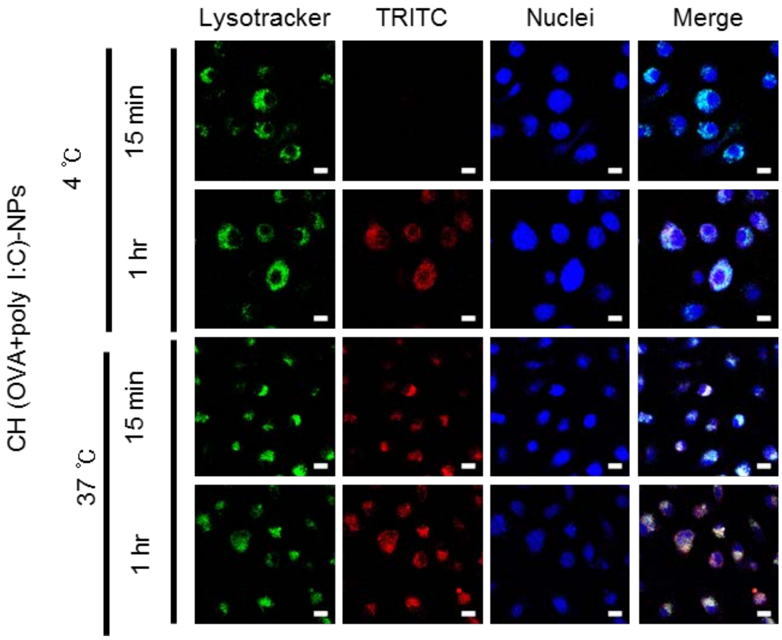

Figure S4. A photograph of Intracellular delivery of CH (OVA+poly I:C)-NPs into DCs at different temperature. Red: TRITC-labeled OVA. Blue: nuclei. Scale bar: 10 μm.

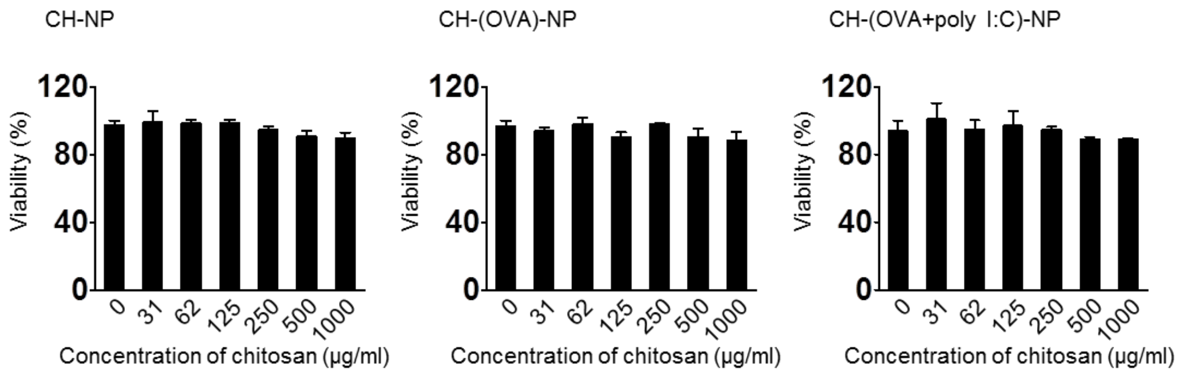

Figure S5. Cell viability following treatment with increasing concentration of chitosan for 24 h incubation. The data are represented as a mean ± S.D. (n=3).

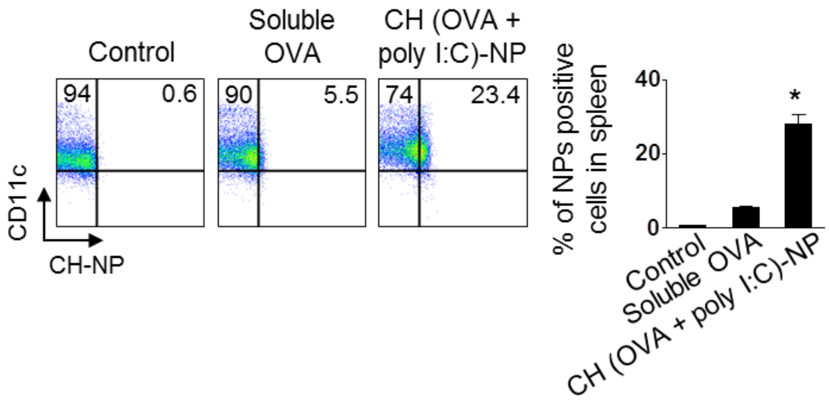

Figure S6. Migration of DCs containing CH (OVA+poly I:C)-NPs to the spleen. Mouse splenocytes were collected and analyzed by flow cytometry for the DCs (stained with anti-CD11c antibody) and CH (OVA+poly I:C)-NPs labeled with TRITC (*p < 0.001). Error bars represent s.e.m

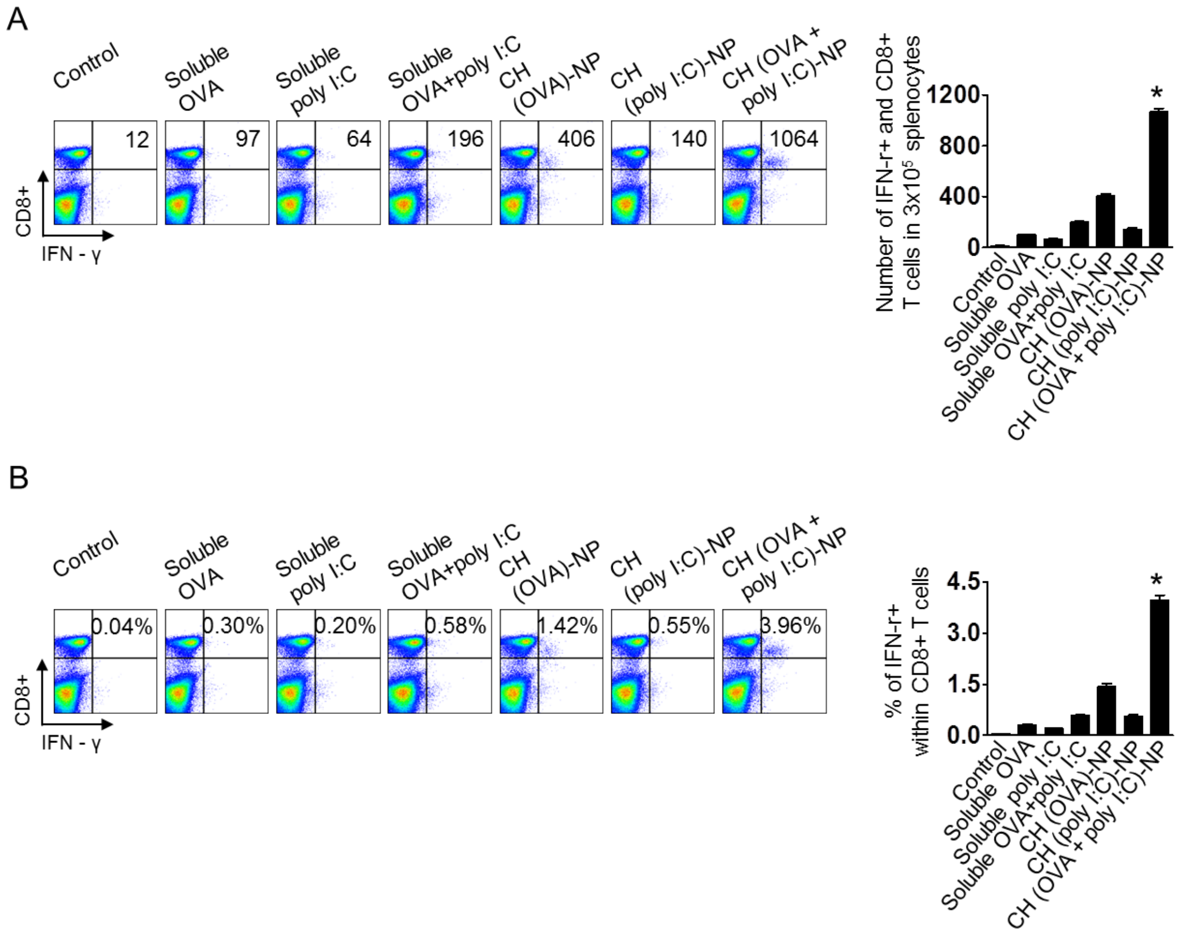

Figure S7. Cytotoxic CD8+ T cell activation was assessed in the splenocytes of the immunized mice by flow cytometric analysis for cells positively stained with anti-CD8 and anti-IFN-γ antibodies. Mice were injected PBS (i.p.) as a control, soluble OVA, soluble poly I:C, soluble OVA+poly I:C, CH (OVA)-NPs, CH (poly I:C)-NPs, or CH (OVA + poly I:C)-NPs via the s.c. route into mice (n = 5 mice per group). (**A**) Number of IFN-γ+ and CD8+ T cells in splenocytes. (**B**) % of IFN-γ+ within CD8+ T cells. The bar graph depicts the number of CD8+ T cells (*p < 0.001). Error bars represent s.e.m.

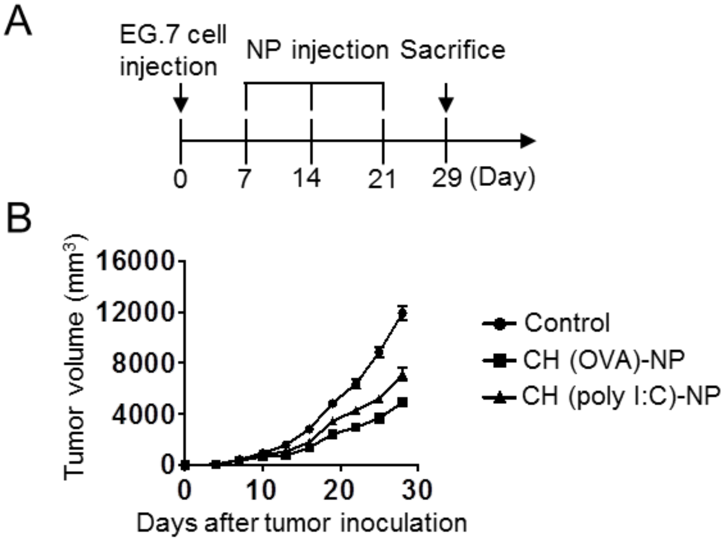

Figure S8. Antitumor efficacy of CH (OVA)-NP or CH (poly I:C)-NP treatment in the EG.7 tumor model. Treatment began 1 week after s.c. injection of tumor cells into the mice. Control, CH (OVA)-NP, or CH (poly I:C)-NP were injected three times at weekly intervals at a dose of 100 µg of OVA and 80 µg of poly I:C via i.p. injection. (**A**) The schedule of the CH-NP-based antitumor treatment. (**B**) Tumor volume after treatment with the various formulations. Error bars represent s.e.m.

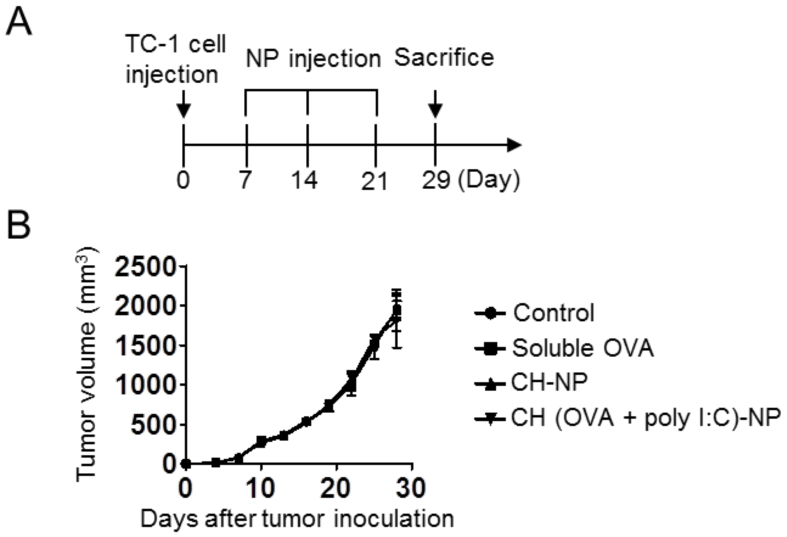

Figure S9. Antitumor efficacy of CH (OVA+poly I:C)-NP treatment in the TC-1 tumor model (OVA-negative tumor) as an irrelevant antigen model. Treatment began 1 week after s.c. injection of tumor cells into the mice. Control, soluble OVA, CH-NPs, or CH (OVA+poly I:C)-NPs were injected three times at weekly intervals at a dose of 100 µg of OVA and 80 µg of poly I:C via i.p. injection. (**A**) The schedule of the CH (OVA+poly I:C)-NP-based antitumor treatment. (**B**) Tumor volume after treatment with the various formulations. Error bars represent s.e.m.

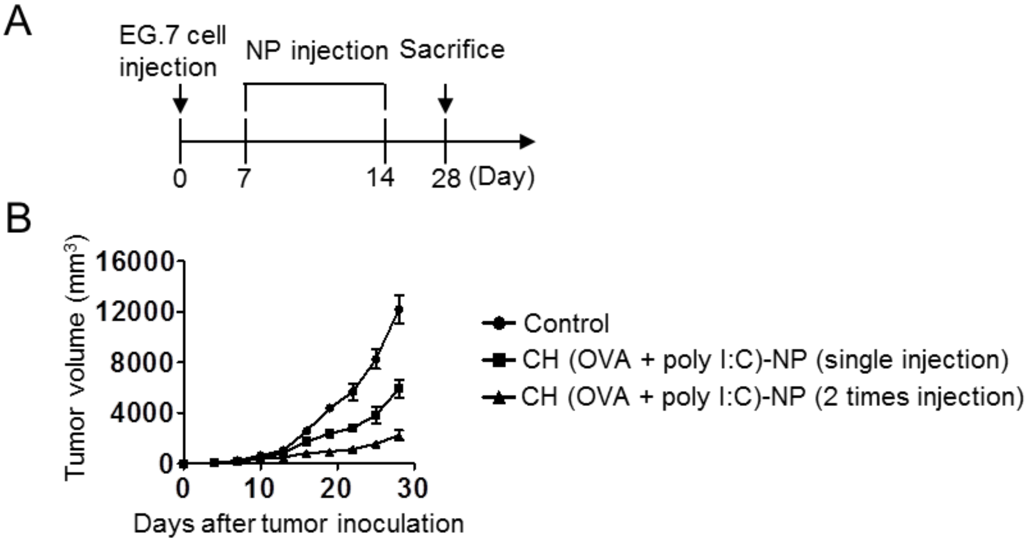

Figure S10. Antitumor efficacy of CH (OVA+poly I:C)-NPs at a different number of injection time points in the EG.7 tumor model. (**A**) The schedule of the CH (OVA+poly I:C)-NP-based antitumor treatment. (**B**) Tumor volume. Error bars represent s.e.m.

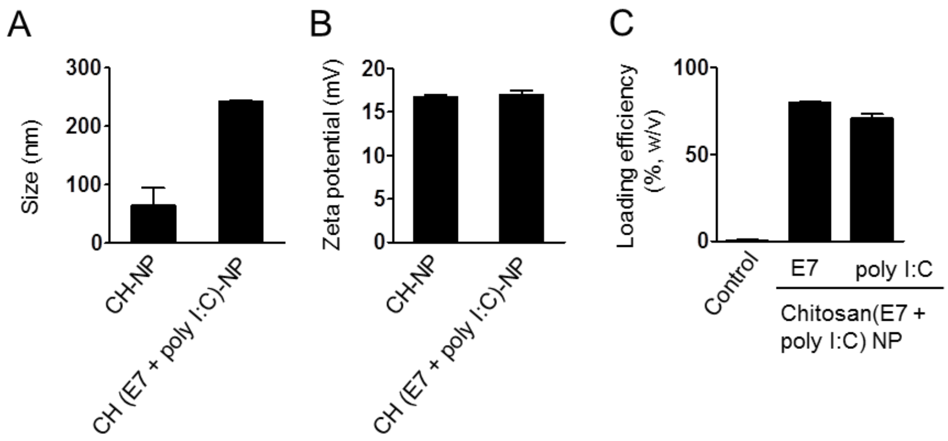

Figure S11. Physical properties of CH (E7+poly I:C)-NPs. (**A**) Size and (**B**) zeta potential of the CH-NPs and CH (E7+poly I:C)-NPs. (**C**) Individual loading efficiency of E7 and poly I:C into CH (E7+poly I:C)-NPs. The data are represented as a mean ± S.D. (n=3).
